# Interdependent Mechanisms for Processing Gender and Emotion: The Special Status of Angry Male Faces

**DOI:** 10.3389/fpsyg.2016.01046

**Published:** 2016-07-14

**Authors:** Daniel A. Harris, Vivian M. Ciaramitaro

**Affiliations:** Developmental and Brain Sciences Program, Department of Psychology, University of Massachusetts Boston, BostonMA, USA

**Keywords:** face perception, contingent adaptation, emotion, visual aftereffects, joint tuning

## Abstract

While some models of how various attributes of a face are processed have posited that face features, invariant physical cues such as gender or ethnicity as well as variant social cues such as emotion, may be processed independently (e.g., Bruce and Young, 1986), other models suggest a more distributed representation and interdependent processing (e.g., Haxby et al., 2000). Here, we use a contingent adaptation paradigm to investigate if mechanisms for processing the gender and emotion of a face are interdependent and symmetric across the happy–angry emotional continuum and regardless of the gender of the face. We simultaneously adapted participants to angry female faces and happy male faces (Experiment 1) or to happy female faces and angry male faces (Experiment 2). In Experiment 1, we found evidence for contingent adaptation, with simultaneous aftereffects in opposite directions: male faces were biased toward angry while female faces were biased toward happy. Interestingly, in the complementary Experiment 2, we did not find evidence for contingent adaptation, with both male and female faces biased toward angry. Our results highlight that evidence for contingent adaptation and the underlying interdependent face processing mechanisms that would allow for contingent adaptation may only be evident for certain combinations of face features. Such limits may be especially important in the case of social cues given how maladaptive it may be to stop responding to threatening information, with male angry faces considered to be the most threatening. The underlying neuronal mechanisms that could account for such asymmetric effects in contingent adaptation remain to be elucidated.

## Introduction

Faces are among the most important social stimuli. Fundamentally adaptive in nature, our ability to perceive the information conveyed by a face can enhance our chances of survival by allowing us to interpret vital, dynamic, and potentially dangerous social information. It has been shown that facial emotions are accurate predictors of future behavior; therefore, appropriately identifying and responding to an angry face may enable us to avoid an ensuing physical threat (Andrew, 1963; Chevalier-Skolnikoff, 1973). The evolutionary relevance of faces is apparent in an infant’s ability to mimic facial expressions just hours from birth (Meltzoff and Moore, 1983) and an adult’s ability to perceive face-like patterns in random stimuli, such as seeing a face on the moon (Liu et al., 2014).

Early models of face processing suggested that the mechanisms for processing different features of a face are independent. Bruce and Young (1986) postulated that structurally different face features, such as gender, identity, emotion, and ethnicity, are processed by separate, distinct, mechanisms in the visual system, which do not interact. Although some neuropsychological (e.g., Calder et al., 1996) and behavioral (e.g., Bruce et al., 1993) evidence supports the Bruce and Young model of *independent* mechanisms for processing of face features, several criticisms of the neuropsychological results had been identified (see Calder and Young, 2005) and subsequent behavioral and neurophysiological studies have found counter evidence, supporting a model of *interdependent* mechanisms, such that different facial features may interact and combine to bias our perception and underlying neuronal processes, (e.g., Atkinson et al., 2005; Ng et al., 2006; Bestelmeyer et al., 2010). One methodology instrumental in determining whether mechanisms are dependent or interdependent is that of adaptation.

Known as the psychophysicist’s electrode, adaptation involves repeated stimulus presentation which reduces neuronal response, biasing the perception of subsequent stimuli in the opposite direction (for a review, see Webster and MacLeod, 2011) and biasing subsequent neuronal activation patterns (e.g., Grill-Spector and Malach, 2001). Adaptation can act on single, simpler, stimulus features, which are fairly low-level, such as orientation or visual motion, such that adapting to upward motion will bias a stationary stimulus to appear to move downward (e.g., Gibson and Radner, 1937; Wade, 1994; Mather et al., 1998; Mather, 2006).

Adaptation can also act on more complex stimulus features. For example, complex aspects of a face can include the encoding of invariant structural statistics, such as the shape of the eyes or nose or the distances between the eyes, or unique facial characteristics which define gender, race or age (e.g., Gibson and Radner, 1937; Kohler and Wallach, 1944; Webster and MacLin, 1999; Muskat et al., 2000; Leopold et al., 2001; Webster et al., 2004; Ackerman et al., 2006; Ng et al., 2006; Russell et al., 2006, 2007). Adapting to an invariant face dimension will bias perception in the opposite direction: adapting to male faces will bias a gender neutral face to appear more feminine.

Complex aspects of a face can also include the encoding of variant features, for example, emotional expressions that are conveyed by dynamically changing structural statistics (e.g., Hsu and Young, 2004; Rutherford et al., 2008; Bestelmeyer et al., 2010; O’Neil and Webster, 2011). The representation of emotional expression is more difficult to conceptualize. For example, what perceptual aftereffect might be expected after adapting to a happy face, given the large number of possible emotions with negative valence? Evidence suggests that a sad expression may be the opposite of a happy expression, such that adapting to a sad face biases a neutral face to be perceived as happier (e.g., Hsu and Young, 2004; Rutherford et al., 2008; but see Juricevic and Webster, 2012). Anti-expressions for each of the basic emotions have been created mathematically to produce a truly opposite spatial configuration and can be selectively adapted (e.g., Juricevic and Webster, 2012). However, such anti-expressions do not fall into clear perceptual emotional categories, such that a face with the spatial configuration of anti-happy does not appear sad or angry, but rather is perceived as emotionally ambiguous.

It has been shown that adaptation to certain features of a face cannot solely be accounted for by retinal adaptation of low-level image statistics. For example, Bestelmeyer et al. (2008) adapted participants to female and male faces or to female and “hyper-female” faces to test if adaptation was specific to stimulus category, gender, or to underlying structural differences. Hyper-female faces were as mathematically different in terms of their structure from female faces as they were from male faces. Their results indicated that adaptation acted at the level of the *category* of gender, female, not at the level of basic structural differences between stimuli, which could be accounted for by low-level retinal adaptation to image statistics. In the case of emotional information as well, adaptation has been found to act at the level of the *category* of emotion, as opposed to low-level structural differences (Etcoff and Magee, 1992; Leopold et al., 2001; Smith et al., 2005).

Given that our experience of a face is not one of single features, it is important to understand how various combinations of face features come to be represented and how they ultimately influence our perception. Researchers aiming to uncover independent versus interdependent processing have often relied on the powerful methodology of *contingent adaptation*, i.e., adapting to a combination of features and looking for evidence of contrasting or opposing aftereffects based on unique stimulus feature combinations. In contingent adaptation two features are paired and always presented together. Given that two different sets of opposing features are presented, if mechanisms for processing are independent there should be no net adaptation since there is equal exposure to opposing conditions. The presence of opposing aftereffects suggests that unique combination of features can be adapted, that they are interdependent and possibly jointly tuned at the neuronal level.

Evidence for contingent adaptation has been found for simple visual features, such as in the classic example of the McCullough effect, where adapting to green–black horizontal gratings and red–black vertical gratings yields orientation specific aftereffects, such that horizontal gratings are biased to be appear redder while vertical gratings are biased to appear greener (McCollough, 1965; Lovegrove and Over, 1972; Wandell et al., 1999). Furthermore, interdependent mechanisms have also been found in the processing of faces across several domains of features. Studies have adapted to a combination of static structural features, such as eye distance and gender, expanded eye distance paired with one gender and reduced eye distance paired with another gender (Little et al., 2005). Here opposite, gender-contingent aftereffects were found as a function of eye distance; responses to faces of one gender were biased toward expanded eye distance, while toward contracted eye distance for the other gender. Evidence also suggests opposite, gender-contingent aftereffects as a function of ethnicity, with contingent adaptation reflected at the neuronal level in areas important for face processing (inferior occipital cortex and the fusiform and cingulate gyrus), suggesting joint neuronal tuning for gender and ethnicity (Ng et al., 2006).

Research investigating interdependent processing for gender and emotion is equivocal. Le Gal and Bruce (2002) found evidence suggesting that gender and emotion, namely anger and surprise, are encoded independently. They adapted the classic Garner (1974) speeded two-choice classification task, to identify the extent to which stimulus dimensions interact, are integral versus separable. They found no difference in reaction times, suggesting that participants could attend independently to either dimension, gender, or emotion, such that processing gender did not interfere with processing emotion. Limitations of the Garner selective attention task have been highlighted in the past (reviewed in Bestelmeyer et al., 2010) and are beyond the scope of the current paper. However, it should be noted that although reaction time differences in this paradigm are taken to imply evidence for interdependent processing, reaction time differences can also arise from differences in the discriminability of stimulus dimensions even if stimulus dimensions are not encoded in an interdependent manner. More recently, Bestelmeyer et al. (2010) used a contingent adaptation paradigm and found evidence suggesting gender and emotion, anger and fear, are encoded interdependently. Opposing perceptual aftereffects were observed after adapting to male-fearful and female-angry or male-angry and female-fearful faces, suggesting that gender and emotion could be encoded in an interdependent manner, since no overall perceptual aftereffects should have been observed if these two dimensions of a face were encoded independently (see below for an elaboration).

In the present study, we not only tested whether mechanisms for processing gender and emotion are interdependent or independent, but also whether such are complementary across emotional categories. We use a contingent adaptation paradigm to investigate mechanisms for face processing along the happy–angry continuum. In contrast to much previous work, we consider complementary conditions of contingent adaptation for gender and emotion, adapting to angry females and happy males in Experiment 1 and the complementary condition, happy females and angry males, in Experiment 2.

**FIGURE 1:**
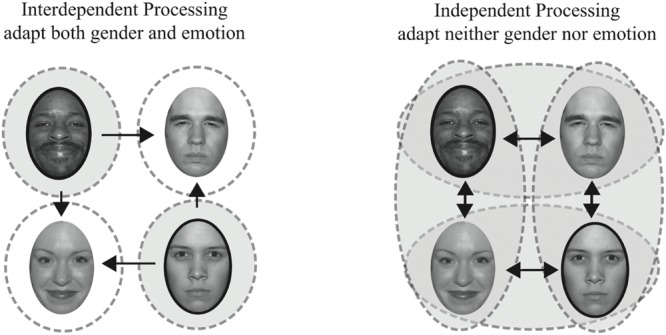
**Interdependent vs. independent processing of emotion and gender.** On the left, dashed circles represent mechanisms selective for both gender and emotion, an interdependent processing model. On the right, dashed ovals represent mechanisms selective for gender or emotion, an independent processing model. The shaded areas reflect what would be adapted in our Experiment 1, where we adapt to angry female and happy male faces. Arrows indicate hypothetical adaptation aftereffects for each model, with the longer, horizontal, arrows indicating the adaptation effects we quantified.

We tested a unique combination of face features, gender and emotion (happy versus angry), as highlighted in **Figure 1** (adapted from Ng et al., 2006). A sample of the contingent pairs of face features adapted in Experiment 1, happy male and angry female faces, are shown outlined in dark ovals. Repeated exposure to these unique feature combinations should yield no net adaptation if features are represented independently since subjects are adapted with equal frequency to male and female faces and to angry and happy faces (illustrated on the right). Repeated exposure to these unique feature combinations should yield adaptation effects in different, opposing, directions if male and female faces are being processed interdependently, such that adaptation to female faces is contingent on one emotion and adaptation to male faces is contingent on another emotion (illustrated on the left). In such a scenario, our Experiment 1, adapting to female angry faces should bias female faces to be perceived as happier, while concurrent adaptation to male happy faces should bias male faces in the opposite direction, to be perceived as angrier.

## Materials and Methods

### Participants

Participants were recruited from the University of Massachusetts Boston community of undergraduate and graduate students via email or posted flyers. A total of 33 participants completed Experiment 1 (18 females, mean age = 23 years, SEM = 0.712, range = 18–33, with no significant difference in age between male and female participants, *p* = 0.31). Experiment 2 was completed by an additional, and different sample, of 33 participants (18 females, mean age = 24 years, SEM = 1.486, range = 18–64, with no significant differences in age between male and female participants, *p* = 0.40). An additional 10 participants completed Experiment 1 but had to be excluded for the following reasons: an insufficient number of completed trials, less than 45 trials per condition (5), biased responses, where subjects selected the wrong emotion for unambiguous faces (80% morphs) more than 50% of the time (2), or a Point of Subjective Equality (PSE; defined in more detail in Data Analysis section) exceeding 2 standard deviations of the mean PSE (3). An additional seven participants completed Experiment 2 but had to be excluded for the following reasons: an insufficient number of completed trials (2), biased responses (4), or experimenter error (1).

All participants reported normal or corrected-to-normal vision and gave informed consent. Participants were compensated monetarily or allotted extra credit in accepted undergraduate psychology courses. This study was approved by the University of Massachusetts Boston Institutional Review Board.

### Stimuli

**FIGURE 2:**
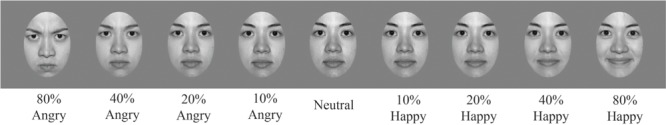
**Sample stimuli.** Examples of a sample female face from the NimStim database, morphed along an emotional continuum, in steps ranging from 80% angry to neutral to 80% happy. Morph steps were chosen to optimize sampling where the dynamic range should be greatest, close to the ‘neutral’ face defined by the NimStim database. All faces were gray scaled and presented on a gray background.

Adapting stimuli consisted of 30 face images (15 female and 15 male; 20 Caucasian, 3 Asian, and 7 Black). Faces were chosen from the NimStim face database (Tottenham et al., 2009). This database includes faces rated and scored for their validity of emotional expression (Tottenham et al., 2009). We only included faces from the NimStim face database with validity ratings of 75% or higher for happy and angry expressions for our set of adapting stimuli. All faces were gray-scaled to 50% and were embedded within a gray oval to reveal only the most emotionally relevant face features, with distracting stimuli such as clothing and hair artifacts occluded (see **Figure 2**).

**FIGURE 3:**
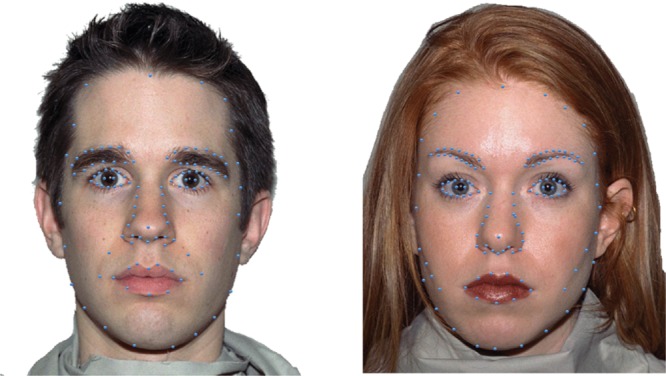
**Sample points for morphing faces.** Faces from the NimStim face database were morphed using MorphMan, by placing points on prominent face features (sample a male face on **Right** and female face on **Left**). Starting points on neutral face images, shown here, were mapped onto end points in the complementary expressive face (100% happy and also 100% angry).

Test stimuli consisted of eight faces (four female and four male; five Caucasian, two Asian, and one Black). Test images were also selected from the NimStim face database (Tottenham et al., 2009) using the same criterion as mentioned for adapting stimuli. Test images were morphed using the MorphMan software package (STOIK Imaging, Moscow, Russia). A fully affective face was morphed with its complementary neutral to simulate an emotional continuum ranging from 80, 40, 20, to 10% angry as well as from 80, 40, 20, to 10% happy (see **Figure 2** to see the angry to happy morphs for a sample female face, top panel, and male face, bottom panel). Morphing was performed by placing points on prominent face features: eyebrow ≈ 28 points; eyes ≈ 30 points; nose ≈ 14 points; mouth ≈ 22 points; face contour ≈ 18 points. Sample faces showing how points were selected for morphing prominent face features are shown in **Figure 3**.

### Apparatus

Face stimuli were presented on a Nexus cathode ray-tube monitor with participants seated 45 cm away from the monitor, positioned on a chin and forehead rest to maintain constant viewing distance. Face stimuli were 595 × 595 pixels and subtended a viewing angle of 19.8 degrees. Stimuli were presented using MATLAB and the psychophysics toolbox (Brainard, 1997; Pelli, 1997). Manual responses were recorded via button press. Participants wore noise-canceling headphones to minimize ambient noise.

### Experimental Procedures

Before the start of the experimental session outlined below, each subject was familiarized with the sequence of events in time and practiced on several trials where they heard an auditory alerting cue, were presented with a blank oval for 1 s, followed by a question mark presented for 1.5 s, during which time they had to arbitrarily respond via button press. A minimum of four practice trials were completed, with subjects given more practice if they failed to understand instructions.

#### Stimulus Sequence for Baseline Condition

**FIGURE 4:**
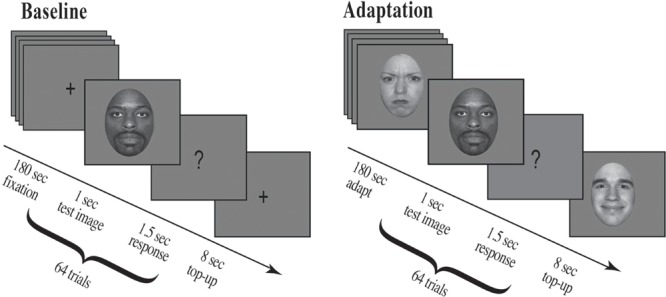
**Experimental procedure.** Participants were run in a two-alternative forced choice task and had to judge a series of randomly presented morphed test faces as either happy or angry (eight unique face identities, four male and four female; each unique face morphed along an emotional continuum from angry to neutral to happy). For the Baseline condition, shown on the left, a fixation cross appeared at screen center (180 s), which participants were asked to fixate. This was followed by a brief auditory cue (500 Hz) which alerted participants to an upcoming stimulus, a test face. The test face appeared for 1 s followed by a question mark (1.5 s), during which time participants had to judge the previously displayed test face. After each question mark a fixation cross appeared at screen center (8 s). For the Adaptation condition, shown on the right, a series of randomly selected adaptation faces appeared at screen center (each face for 1 s for a total of 180 s; 30 possible unique face identities, 15 male and 15 female, the original NimStim sample faces, defined as 100% emotional). This example depicts stimuli for Experiment 1, where participants were adapted to angry females and happy males. Adaptation was followed by a brief auditory cue (500 Hz) which alerted participants to an upcoming stimulus, a test face. The test face appeared for 1 s followed by a question mark (1.5 s), during which time participants had to judge the previously displayed test face. After each question mark an additional series of eight randomly selected adaptation faces were presented for 1 s each (Top-up Adaptation). In Experiment 1, all adapting female faces were angry and all adapting male faces were happy, whereas in Experiment 2, all adapting female faces were happy and all adapting male faces were angry.

For the first 180 s, participants fixated a cross presented at screen center. At the end of this period an auditory cue (500 Hz) alerted participants to an upcoming stimulus, a test face, which appeared for 1 s followed by a question mark for 1.5 s. Participants were asked to make a forced choice decision and judge the previously displayed test face as either happy or angry during the time the question mark was displayed. They pressed the ‘z’ key for faces judged happy and the ‘x’ key for faces judged angry. After each test face a fixation cross was presented at screen center for 8 s (see **Figure 4** for details).

A total of 64 test images were presented: eight neutral faces, eight face morphs for each of the angry and happy morphs at 10, 20, and 40%, and four face morphs for each of the angry and happy morphs at 80%. The order of test image presentation varied randomly across trials. Participants were instructed to fixate gaze at screen center throughout the trial, but eye position was not monitored.

#### Stimulus Sequence for Adaptation Condition

For the first 180 s, participants fixated screen center as a series of 180, randomly chosen, adapting faces (100% emotion) were presented, each for 1 s. At the end of this adaptation period an auditory alerting cue (500 Hz) indicated an upcoming stimulus, a morph test face, which appeared for 1 s followed by a question mark for 1.5 s. Participants were asked to make a forced choice decision, to judge the previously displayed test face as either happy or angry during the time the question mark was displayed by pressing the ‘z’ key for faces judged happy and the ‘x’ key for faces judged angry. After each test image a series of 8, randomly chosen, adapting faces (100% emotion) were presented for 1 s each for a total top-up adaptation period of 8 s (see **Figure 4** for details).

As in the baseline condition, a total of 64 test images were presented: eight neutral faces, eight face morphs for each of the angry and happy faces for 10, 20, and 40% morphs, and four faces morphs for each of the angry and 80% morphs. As in baseline, the order of test image presentation varied randomly across trials. Participants were instructed to fixate gaze at screen center throughout the trial, but eye position was not monitored.

The duration and sequence of events in time were similar for the baseline and adaptation condition to equate for subject fatigue as a function of time on task within a condition. The only difference between conditions was the presentation of a blank screen with a fixation cross (in baseline) versus a face at 100% emotion (in adaptation) during the 180 s start of each session and during the 8 s top-up adaptation period. For Experiment 1, adapting faces consisted of angry female and happy male faces at 100% emotion (the original NimStim sample faces). For Experiment 2, adapting faces consisted of happy female and angry male faces at 100% emotion (the original NimStim sample faces). Each experiment consisted of one baseline condition and one adapt condition, each lasting roughly 18 min, with a 5 min break between.

### Data Analysis

For the analyses described below, trials for which participants did not make a judgment in the allotted time were excluded from further analysis. Out of the possible 64 trials total, participants completed an average of 58.12 baseline trials (SEM: 0.75; range: 50–64) and 60.52 adapt trials (SEM: 0.496; range: 50–64) for Experiment 1 and 57.91 baseline trials (SEM: 0.885; range: 48–64) and 60.12 adapt trials (SEM: 0.593; range: 52–64) for Experiment 2. Data for Experiment 1 reflects a total of 1,918 baseline trials and 1,997 adapt trials across 33 subjects, while data for Experiment 2 reflects a total of 1,911 baseline trials and 1,984 adapt trials across 33 subjects.

#### Quantifying Changes in the Point of Subjective Equality

During the baseline condition, we aim to capture each participant’s intrinsic bias in the identification of emotion in male and female faces. We calculate each participant’s unique neutral point, or PSE, by determining which face morph the participant is equally likely to judge as happy or angry. The PSE allows us to determine the mathematical face morph, even if never presented, that is judged emotionally ambiguous, at chance levels or 50% for judging a face happy or angry. We measure the strength of adaptation separately for female and male faces by quantifying changes in judgments of the morph judged emotionally neutral at baseline, i.e., how much more or less happy the female and male face morph at the PSE is judged after adaptation.

**FIGURE 5:**
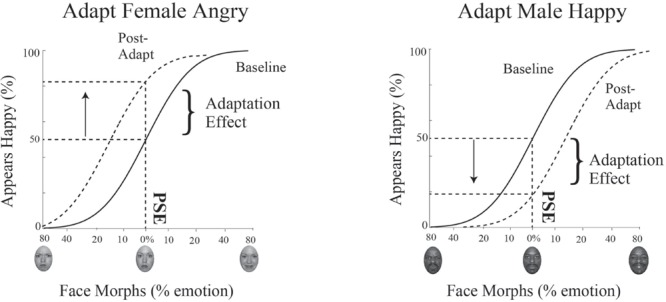
**Psychophysics data fitting and predictions.** The *x*-axis represents the morphed face continuum and the *y*-axis represents the percentage of happy responses. Data was fit using a cumulative normal. The solid black line depicts the fit to baseline data. From this fit, we determined each participant’s unique neutral point, or PSE, the morph supporting 50% happy judgments, where the face was equally likely to be judged happy or angry. The dotted black line depicts the fit to post-adaptation data. We quantified the adaptation effect by determining how much happier or angrier the face morph judged neutral at baseline (at the PSE) was post-adaptation, indicated by the arrow. After adaptation to angry female faces (and happy male faces, not shown), an interdependent model would predict the female faces judged neutral at baseline would be judged happier (depicted as an upward shift and shown on the **Left**). After adaptation to happy female faces (and angry male faces, not shown), an interdependent model would predict the face judged neutral at baseline would be judged angrier (depicted as a downward shift and shown on the **Right**).

To determine the PSE for male and female faces, the dataset for the baseline condition and for the adaptation condition is subdivided based on the gender of the face morph and each dataset is then fit with a psychometric function. Data is plotted such that the *x*-axis is the morph continuum for a given gender and the *y*-axis is the percentage of happy responses for each face morph. This data is fit using a cumulative normal function. To quantify the shift in the PSE post-adaptation, we determine the change in the judgment for the face morph judged neutral at baseline, i.e., the percent change in judgments of happy along the *y*-axis (see **Figure 5**).

If mechanisms for processing gender and emotion are interdependent, we expect adaptation to angry female and happy male faces to produce simultaneous aftereffects in opposite directions, contrastive aftereffects, with male faces judged happier and female faces judged angrier. The lack of such contingent adaptation, no contrastive aftereffects for male and female faces, would suggest independent processing for gender and emotion. A concrete example of expected results for contingent adaptation in our paradigm for Experiment 1 is illustrated in **Figure 5**. In Experiment 1 participants are adapted to angry female and happy male faces. Post-adaptation, if the 0% female morph was judged neutral at baseline, this same morph should be viewed as happier (a positive shift, as depicted by the upward arrow in **Figure 5** on the left), while, if the 0% male morph was judged neutral at baseline, this same morph should be viewed as angrier (a negative shift, as depicted by the downward arrow in **Figure 5** on the right). Evidence for contingent adaptation is expected to yield complementary effects in the opposite direction in Experiment 2, where subjects are simultaneously adapted to happy female and angry males faces.

## Results

**FIGURE 6:**
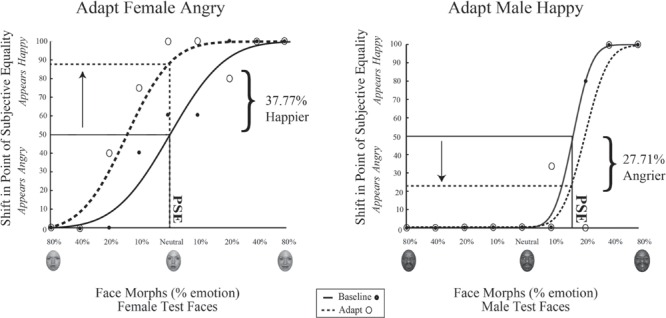
**Psychophysical results for a sample participant.** Experiment 1 single participant data. Plotted on the *x*-axis is the morphed face continuum, angry to happy. The *y*-axis represents the percentage of trials the participant judged a given face morph as happy. Solid black lines represent fits to the baseline data, while dotted black lines represent fits to the post-adaptation data. After adaptation to angry female and happy male faces, the baseline PSE face morph for female faces was judged 24.03% happier **(Left)** while the baseline PSE face morph for male faces was judged 29.69% angrier **(Right)**.

Data for a representation single participant in Experiment 1 is shown in **Figure 6**. The range of face morphs for which subjects had to make a judgment is plotted along the *x*-axis for female faces on the left and male faces on the right, with the convention of angry emotions to the left of zero and happy emotions to the right of zero. For this subject, the female face morph judged happy 50% of the time (baseline PSE) was at –0.0376% angry. This same female morph was judged 37.77% happier (PSE shift) after adaptation to female angry faces. Meanwhile, the male face morph judged happy 50% of the time (baseline PSE) was at 17.868% happy. This same male morph was judged 27.71% angrier (PSE shift) after adaptation to male happy faces. Thus, simultaneous adaptation to angry females and happy males led to contrastive aftereffects in opposite directions, supporting interdependent mechanism for processing face gender and emotion.

**FIGURE 7:**
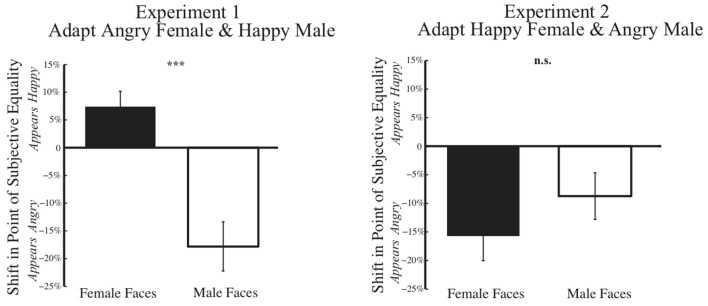
**Psychophysical results post-adaptation.** Data depicting the change in judgment of the face morph judged emotionally neutral at baseline (Baseline PSE) are depicted for female and male test faces for Experiment 1 **(Left)** and Experiment 2 **(Right)**. The *x*-axis represents the gender of the test face, while the *y*-axis represents the percent change in judgment of the unique face judged neutral at baseline for each subject. Thus, each subject’s data is normalized based on initial biases in judging a face. Data show the mean change in judgment (±SEM across subjects). In Experiment 1 **(Left)**, post-adaptation to angry female and happy male faces, female faces are judged happier, an overall positive shift, while male faces are judge angrier, an overall negative shift. In Experiment 2 **(Right)**, such contrastive aftereffects are not found. Rather, post-adaptation to happy female and angry male faces, both female and male faces are judged angrier an overall negative shift (^∗^*p* < 0.05; ^∗∗∗^*p* < 0.001).

A summary of results from Experiment 1 across our 33 subjects is shown on the left in **Figure 7**. In a 2 × 2 mixed variable design, where the dependent variable was the post-adaptation percent shift in judgments of the PSE face at baseline, the independent within-subject variable was the gender of the face morph (male vs. female), while the between-subject independent variable was the gender of the participant, a MANOVA revealed a significant main effect of gender of the morph test face [*F*(1,31) = 28.504, *p* = 0.000, η^2^= 0.479, and observed power = 0.999]. After adaptation to angry female and happy male faces, participants showed post-adaptation shifts in opposite directions, with the female face at the PSE judged happier and the male face at the PSE judged angrier. Interestingly, we found no significant effect of gender of the participant [*F*(1,31) = 0.391, *p* = 0.536, η^2^= 0.012, and observed power = 0.093].

A summary of results from Experiment 2 across our 33 subjects is shown on the right in **Figure 7**. Using the same 2 × 2 mixed variable design outlined for Experiment 1, MANOVA revealed no evidence of contingent adaptation [*F*(1,31) = 2.054, *p* = 0.162, η^2^= 0.062, and observed power = 0.284]. We found no significant difference in adaptation based on gender of the morph test face *F*(1,31) = 2.504, *p* = 0.162, η^2^ = 0.062, observed power = 0.284. Nor did we find any significant effect of the gender of the participant [*F*(1,31) = 1.942, *p* = 0.173, η^2^= 0.059, and observed power = 0.272]. Overall, the results of Experiment 2 do not support interdependent mechanisms for face processing, which would have predicted contrastive aftereffects.

*Post hoc* analyses across conditions from Experiments 1 and 2, to test if the mean magnitude of the adaptation effect (irrespective of direction) was weaker when adapting to angry versus happy faces, found a trend for comparatively weaker adaptation for angry versus happy faces, irrespective of the gender of the face [*t*(32) = -1.55, *p* = 0.063].

**FIGURE 8:**
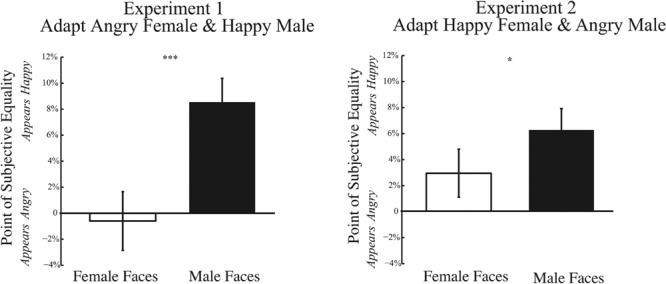
**Psychophysical results at baseline.** Data depicting baseline biases for female and male test faces are shown for Experiment 1 **(Left)** and Experiment 2 **(Right)**. The *x*-axis represents the gender of the test face, while the *y*-axis represents the baseline point of subjective quality. Data shows the mean PSE at baseline (±SEM across subjects). In both Experiments 1 and 2, the baseline PSE for male test faces is significantly more positive (happier) than for female test faces, indicating that male faces require more happiness to be perceived as neutral and are biased to be perceived more negatively (^∗^*p* < 0.05; ^∗∗∗^*p* < 0.001).

One possible explanation for weakened adaptation could arise from biases in perceiving anger in a face at baseline. Our quantification of adaptation is normalized by results at baseline. If the baseline PSE is already biased, this might restrict the dynamic range over which adaptation could alter responses, minimizing adaptation effects specifically for angry faces. **Figure 8** shows the baseline PSE for Experiments 1 and 2, on the left and right, respectively. Across both Experiments, we find a significant main effect of gender of the morph test face. For both Experiments 1 and 2, baseline PSE is significantly more positive for male compared female test faces [Experiment 1: *t*(32) = 3.500, *p* = 0.001; Experiment 2: *t*(32) = 2.118, *p* = 0.042]. These results indicate that, at baseline, the PSE is significantly more positive for male compared to female faces, indicating that a male face must contain more happiness to be perceived as emotionally neutral. Thus, there is a bias in perceiving male faces more negatively from the outset. Although baseline biases could account for the trend for weaker adaptation effects to angry male faces, they cannot account for the wrong direction of this adaptation effect, where adaptation to angry male faces should bias the percept of neutral male faces to be happier, a net positive shift. Nor can baseline biases account for the trend for weaker adaptation to angry female faces, since no baseline bias was observed for female faces.

## Discussion

We used a classic method, contingent adaptation, to test if mechanisms for processing the gender and emotion of a face were interdependent or independent. Our task design was optimized for adaptation to the *categories* of emotion and gender rather than to unique face identities of a particular gender and emotion given that we used 30 unique face identities during adaptation and a subset of eight unique face morph identities during test. Our results from Experiment 1, where we adapt to angry female and happy male faces, provide evidence for contrastive perceptual aftereffects: after adaptation, female faces at the PSE are judged happier while male faces at the PSE are judged angrier. These results are what would be predicted if mechanisms for processing gender and emotion are interdependent, corroborating and extending previous work finding evidence for interdependent processing among different combinations of face features, such as gender and emotion, (Hsu and Young, 2004; Webster et al., 2004; Bestelmeyer et al., 2008, 2010) gender and ethnicity (Ng et al., 2006; Bestelmeyer et al., 2010), and even gender and structural features, such as eye spacing (Little et al., 2005).

Contrary to the predictions of interdependent mechanisms, our results from Experiment 2, where we adapted to happy female and angry male faces, failed to provide evidence for contrastive perceptual aftereffects. Thus, although female faces at the PSE were judged angrier after adaptation, male faces at the PSE were not judged happier after adaptation. Interestingly, it is not the case that female test faces show no net adaptation effect, as predicted from an independent mechanism for processing face gender and emotion. Nonetheless, the unique combination of angry male faces confers a special processing status, rendering such a combination of facial features resistant to classic contingent adaptation effects.

Across experiments, we found a trend, albeit insignificant, for overall weaker adaptation to angry compared to happy faces, for both female and male faces, corroborating and extending previous neurophysiological evidence for weaker adaptation to anger (e.g., Gerlicher et al., 2014) and extending previous behavioral evidence that male faces are biased to be perceived as angry (for example, Bayet et al., 2015). The biases we find at baseline, while reducing the dynamic range over which adaptation can act, cannot account for our failure to find opposite adaptation effects for angry male faces compared to happy male faces.

How is it that the failure of contingent adaptation effects for one condition, but not its complement, have not been noted before? We tested complementary conditions, something not always considered. In addition, even when complementary conditions have been considered, analytic methods may have obscured results. For example, Bestelmeyer et al. (2010) tested complementary stimulus pairs in their contingent adaptation paradigm, angry females and fearful males or fearful females and happy males; yet, they collapsed across gender of the test images to examine the effect of fearful versus angry. Thus, biases as a function of face gender may have been obscured. In addition, their study focused on negative, threatening, and emotions. In our experimental paradigm, we separate out effects based on the gender of the test image and focus on a negative and positive emotion. The unique combination of gender and emotion we chose allowed us to uncover the special status of angry male faces at the behavioral level. Our behavioral findings are in line with neuroimaging results, which find evidence for weaker neuronal adaptation in face selective areas when adapting to angry male faces (Gerlicher et al., 2014; although see Suzuki et al., 2011, who found the weakest fMRI adaptation effects for happy faces).

Furthermore, it is unlikely that the gender of the participant could account for the asymmetry in contingent adaptation effects between Experiments 1 and 2. The same number of female and male participants were included in both experiments and gender of the participant was not a significant factor in either of our experiments. Though we did not observe significant differences in our emotional adaptation paradigm, we acknowledge that gender differences in face perception have been highlighted in the literature. For example, compared to males, females exhibit a superior ability for remembering faces (Goldstein and Chance, 1971; Guillem and Mograss, 2005) and detecting facial emotions (Hall and Matsumoto, 2004). In a morphed face task, Montagne et al. (2005) found that female participants were both faster and more accurate than male participants in judging emotions in video clips of faces morphing from neutral to a fully affective face (happiness, sadness, anger, disgust, fear, or surprise). We cannot exclude the possibility that the lack of an influence of gender of the participant in our experiments could in part be attributed to low statistical power.

One possible explanation for our results is that angry male faces are so emotionally salient that they are resilient to adaptation effects. Such resilience to adaptation may arise from socialization, and should be especially true for negative emotions, such as anger, given that they serve as a cue to potentially threatening environments for which we need to maintain alertness and responsiveness. The selective pressure for fast responsiveness to negative emotional information, may have driven the development of perceptual and cognitive mechanisms with enhanced processing for negative emotions. In fact, evidence suggests that reaction times to threatening, negative, information are faster (Adams et al., 2010; Tamietto and de Gelder, 2010). Previous studies have highlighted factors that socialize association between gender and emotion (for a review, see Tay, 2015). It has been shown that males express anger more often than females in social situations (Fabes and Martin, 1991). Considering that displays of facial emotion have been shown to be accurate predictors of future behavior, it would be advantageous to associate maleness with anger (Andrew, 1963; Chevalier-Skolnikoff, 1973).

The special status for angry male faces in our study may be the result of two mechanisms, one for increased salience, or attention, and one for weakened adaptation. Both mechanisms play a role in optimizing visual performance, adaptation via optimizing sensitivity for novel over familiar stimuli and attention via optimizing sensitivity to behaviorally relevant over irrelevant stimuli. Such optimization in performance is often accomplished via an optimization of the dynamic range for which the sensory system is tuned, for example adaptation can shift tuning curves for contrast sensitivity and attention can alter contrast tuning curves via changes in contrast gain or response gain.

The allocation of attention to faces has been shown to be biased as a function of emotional valence (e.g., Eastwood et al., 2001; Vuilleumier, 2002). For example, threatening faces tend to capture and hold our attention (Koster et al., 2004) and attention can be captured more readily by angry male faces compared to positive or neutral faces (e.g., Bannerman et al., 2010; Feldmann-Wüstefeld et al., 2011; Huang et al., 2011; see Holmes et al., 2009 for an example at the level of physiology). Moreover, adaptation has been shown to be biased by emotional valence. For example, adaptation to face identity has been shown to decrease as a linear function of the negative valence of the emotional expression of the face (Gerlicher et al., 2014). Finally, previous studies suggest that attention and adaptation can interact. For example, Rezec et al. (2004) found that attending to a low-level visual feature, such as visual motion, can yield an “adaptation gain,” with enhanced adaptation to visual motion for attended stimuli relative to weaker adaptation under conditions of passive viewing (for an example for motion aftereffects, see also Lankheet and Verstraten, 1995; Alais and Blake, 1999; for contrast aftereffects, see Ling and Carrasco, 2006). Neuronal evidence suggests that attention to low level visual features, such as orientation (e.g., Murray and Wojciulik, 2004), may increase the adaptability of neurons, with more pronounced effects of attention on adaptation as one ascends the visual hierarchy (see news and views by Boynton, 2004). Attention has also been shown to increase the gain of adaptation for more complex stimuli, such as faces. For example, Rhodes et al. (2011) found that face identity and figural (distortion) aftereffects were enhanced by attention.

Given the above, one might have expected heightened adaptation for more salient emotional faces; threatening faces should preferentially capture attention and strengthen the effects of adaptation. However, we observed the opposite patterns of results, heightened sensitivity to angry male faces after repeated exposure to angry male faces. Interestingly, although we found the expected, complementary and opposing aftereffects for female faces, adaptation tended to be weaker, albeit not significantly, for angry relative to happy female faces, suggesting weakened adaptation for more salient threatening emotional information. This pattern of adaptation results for female faces is expected if attention acts after adaptation. For simple visual stimuli, attention and adaptation can be separate and independent mechanisms, such that attention can act after adaptation to overcome the initial effects of adaptation and restore contrast sensitivity (Pestilli et al., 2007) and alter perceived speed (Anton-Erxleben et al., 2013). For more complex and emotional visual stimuli, it remains to be seen if attentional biases to select emotions can serve as a mechanism to overcome initial adaptation and maintain emotional sensitivity, or if weakened adaptation is accomplished through some other mechanism. While interesting, we highlight that the above mentioned effects were not significant, and only a trend, and that such an account cannot explain the pattern of results we observe for male faces.

In order to tease apart the specific contributions of attention versus adaptation in responses to emotional faces, future research could (1) adapt to less salient angry male faces (2) parameterize anger to create true equivalence in salience between male and female faces for a given emotional valence, especially for threatening emotional stimuli or (3) determine if mechanisms of adaptation might be influenced by the perceived maleness of a given face as the expression of an angry emotion can bias gender neutral faces to appear more masculine (e.g., Hess et al., 2009). Unlike in our current paradigm, the allocation of attentional resources would need to be explicitly controlled, especially the timing of the re-allocation of attentional resources, before versus after adaptation.

In sum, our results suggest that finding evidence in support of *interdependent* versus *independent* mechanisms for processing emotion and gender may depend on the unique combination of face features being considered. Whether or not there is a special status imparted to emotional information in general, threatening emotional information in particular, or whether other unique combinations of facial features might also be resilient to adaptation, remains to be determined as does the development of such potential biases in how combinations of face features are processed and their neuronal underpinnings.

## Author Contributions

Toward this project, DH contributed to stimulus generation, data collection, data analysis and interpretation, and writing of the manuscript, and VC contributed to experimental design, stimulus presentation, data analysis and interpretation, and writing of the manuscript.

## Conflict of Interest Statement

The authors declare that the research was conducted in the absence of any commercial or financial relationships that could be construed as a potential conflict of interest.
